# No Effect of a Self-Help Book for Insomnia in Patients With Obstructive Sleep Apnea and Comorbid Chronic Insomnia – A Randomized Controlled Trial

**DOI:** 10.3389/fpsyg.2018.02413

**Published:** 2018-11-29

**Authors:** Bjørn Bjorvatn, Thomas Berge, Sverre Lehmann, Ståle Pallesen, Ingvild W. Saxvig

**Affiliations:** ^1^Norwegian Competence Center for Sleep Disorders, Haukeland University Hospital, Bergen, Norway; ^2^Department of Global Public Health and Primary Care, University of Bergen, Bergen, Norway; ^3^Centre of Sleep Medicine, Haukeland University Hospital, Bergen, Norway; ^4^Section of Thoracic Medicine, Department of Clinical Science, University of Bergen, Bergen, Norway; ^5^Department of Psychosocial Science, University of Bergen, Bergen, Norway

**Keywords:** OSA, insomnia, AHI, cognitive behavioral therapy, CBTi, sleep apnoea

## Abstract

**Objective:** To compare the effects of a self-help book for insomnia to that of sleep hygiene advice in a randomized controlled trial with follow-up after about 3 months among patients who were diagnosed with obstructive sleep apnea (OSA) and comorbid chronic insomnia, and who were concurrently initiating treatment with continuous positive airway pressure (CPAP).

**Methods:** In all, 164 patients were included. OSA was diagnosed and categorized based on a standard respiratory polygraphic sleep study using a type 3 portable monitor. The self-help book focused on cognitive behavioral therapy for insomnia. The main outcome measure was insomnia severity assessed with the Bergen Insomnia Scale (BIS) and the Insomnia Severity Index (ISI).

**Results:** The scores on the BIS improved significantly from pre-treatment to follow-up in the sleep hygiene advice group (26.8 vs. 21.8) and in the self-help book group (26.3 vs. 22.4). Similarly, the ISI scores were significantly improved in both conditions (sleep hygiene: 17.0 vs. 14.1; self-help book: 16.6 vs. 13.6). No time × condition interaction effects were detected, suggesting that the self-help book did not improve insomnia symptoms more than the sleep hygiene advice.

**Conclusion:** In this randomized controlled trial among patients with OSA and comorbid insomnia who were initiating CPAP treatment, concurrently treating their insomnia with a self-help book did not improve sleep more than sleep hygiene advice. The statistically significant improved sleep at follow-up in both groups is most likely explained by the CPAP treatment.

## Introduction

Obstructive sleep apnea (OSA) is a highly prevalent sleep disorder characterized by breathing pauses, oxygen desaturation, and arousals during sleep (American Academy of Sleep Medicine, 2014). The severity of OSA is usually characterized with the apnea-hypopnea-index (AHI), in which a higher AHI indicates more severe OSA. The disorder typically co-occurs with other sleep related problems, such as insomnia symptoms (Luyster et al., 2010; Bjorvatn et al., 2015). Chronic insomnia is characterized by difficulties initiating or maintaining sleep to such a degree that it causes daytime impairments for a period of 3 months or more (American Academy of Sleep Medicine, 2014). Non-restorative sleep (poor sleep quality) was previously a diagnostic criterion for chronic insomnia, but is no longer part of the diagnostic criteria according to the fifth and latest version of the Diagnostic and Statistical Manual for Mental disorders (DSM-5) (American Psychiatric Association, 2013) and the International Classification of Sleep Disorders-3 (ICSD-3) (American Academy of Sleep Medicine, 2014). As non-restorative sleep is commonly reported by OSA patients, these revised criteria will reduce the prevalence of insomnia among OSA patients. In line with this, we recently reported that the prevalence of chronic insomnia disorder among OSA patients was reduced from 74–79% (depending on OSA severity) using the DSM-IV/ICSD-2 criteria to 44–55% using the DSM-5/ICSD-3 criteria (Bjorvatn et al., 2015).

The relation between OSA and insomnia is likely bidirectional (Sweetman A.M. et al., 2017; Philip et al., 2018). For instance, OSA may disturb sleep and increase the number of nightly awakenings (Krakow et al., 2001; Smith et al., 2004). OSA may therefore cause, exacerbate, or contribute to symptoms of insomnia (Krakow et al., 2001; Sweetman A.M. et al., 2017; Philip et al., 2018). On the other hand, insomnia may exacerbate OSA (Beneto et al., 2009; Sweetman A.M. et al., 2017) and impede sleep apnea treatment (Bjornsdottir et al., 2013; Philip et al., 2018; Wallace et al., 2018). For instance, insomnia may lead to a reduction in upper airway muscle tone via abrupt wake–sleep transitions, as reviewed by Sweetman A.M. et al. (2017). Furthermore, patients with insomnia are typically sensitive to disturbing factors during sleep. Standard treatment of OSA with continuous positive airway pressure (CPAP) may, due to cumbersome feelings of wearing the mask and noise from the machine, be problematic (Bjornsdottir et al., 2012, 2013; Sweetman A.M. et al., 2017). In line with this, two recent studies showed that insomnia symptoms were associated with early quitting of CPAP treatment (Eysteinsdottir et al., 2017) and lower CPAP compliance at 6 month follow-up (Wallace et al., 2018). For these reasons, successful treatment of insomnia may prove to be highly important for OSA patients.

The treatment of choice for chronic insomnia is cognitive behavioral therapy (CBTi) (Riemann et al., 2017). Unfortunately, few therapists offer CBTi, and CBTi is rarely offered in clinics focusing on treatment of OSA. The reasons for this may be that such treatment is time consuming and not easily available (Morin et al., 2005). However, several studies show that self-help therapies may be effective. In a meta-analysis including 10 randomized controlled studies of self-help therapies for insomnia, it was concluded that such interventions provide small to moderate effects (van Straten and Cuijpers, 2009). We have previously shown that a self-help book for insomnia, with focus on CBTi, significantly improved several sleep measures compared to sleep hygiene advice in a randomized controlled trial among patients with chronic insomnia (Bjorvatn et al., 2011). However, that study was conducted among patients without comorbidities. Whether such self-help book also will help OSA patients with comorbid insomnia is currently not known. OSA patients may have lower motivation to follow cognitive behavioral techniques compared to patients with primary insomnia. Studies have for example shown that adhering to CBTi (such as leaving bed) when using a CPAP mask may be challenging (Ong et al., 2017). Still, if such a self-help book would prove to be effective in patients with OSA and comorbid insomnia (so-called COMISA), this patient group could utilize a low-cost and easily available low-threshold treatment alternative to standard treatment modes of CBTi.

Against this backdrop, the main objective of the present study was to compare the effect of standard sleep hygiene advice and a self-help book for insomnia in a randomized controlled trial (RCT) with 3-month follow-up among patients with OSA and comorbid insomnia (diagnosed by the updated DSM-5 diagnostic criteria). The sleep hygiene advice/self-help book was provided concurrently with CPAP initiation. The primary outcome measure was insomnia severity as assessed by validated self-report questionnaires, and the secondary outcome measures were objectively assessed CPAP adherence and sleep apnea severity (AHI), as well as the prevalence of chronic insomnia. We hypothesized that the self-help book would reduce insomnia symptoms more than the sleep hygiene advice, and thereby result in increased CPAP adherence and reduced AHI.

## Materials and Methods

The study was a randomized controlled double-blind efficacy trial comparing two treatments for chronic insomnia: (1) a self-help book “Bedre søvn. En håndbok til deg som sover dårlig” (“Better sleep. A handbook for you who sleep poorly”) and (2) standard sleep hygiene advice. The participants comprised OSA patients with comorbid chronic insomnia (COMISA) who concurrently were initiating treatment with CPAP. The sleep hygiene advice were given on a sheet of paper, and included advice about caffeine, nicotine, food intake, exercise etc. (Table 1). The self-help book (187 pages) was published in 2013 (revised edition) (Bjorvatn, 2013). It covers normal sleep and sleep regulation, how sleep problems are assessed, and describes different causes of poor sleep. The main focus of the book is CBTi which is reviewed in detail in the book. To demonstrate the methods in the most tangible and recognizable manner in the book a typical patient with insomnia is followed through assessment, diagnosis, and treatment of the sleep problems. One aim of the self-help book is that patients with chronic insomnia will be able to carry out the treatment program on their own.

**Table 1:** Sleep hygiene advice.

• Avoid caffeinated drinks during the last hours before bedtime (coffee, tea, cola).
• Avoid smoking/nicotine during the last hours before bedtime.
• Avoid alcohol as a sleep aid.
• Avoid going to bed hungry, but do not consume a heavy meal before bed.
• Keep the bedroom dark, quiet, and with moderate temperature. If necessary, use mask and earplugs.
• Regular exercise is good, but do not exercise during the last hours before bedtime.

**FIGURE 1:**
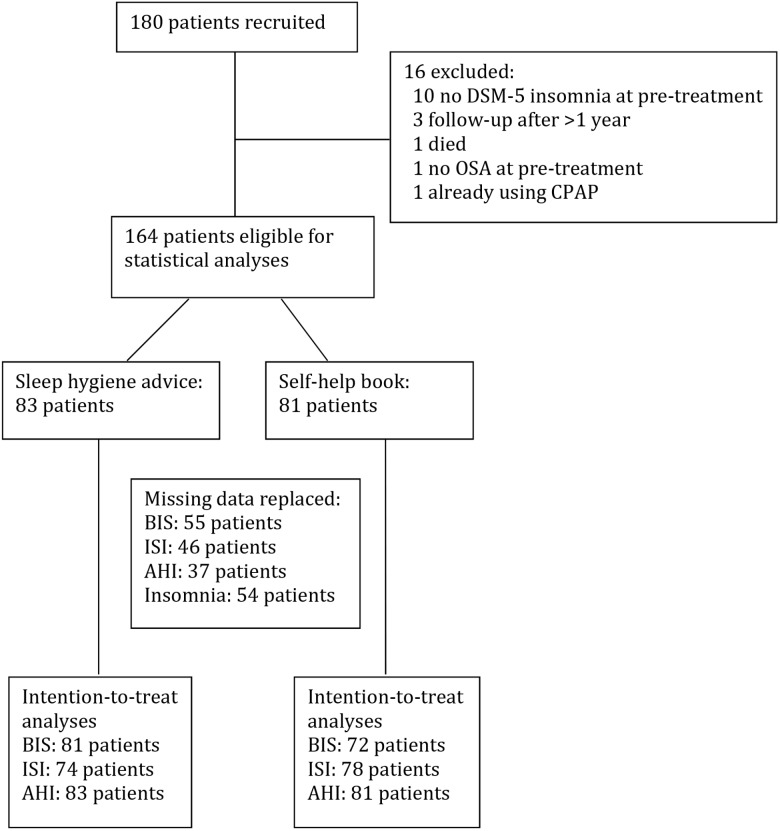
Overview of the participants in the study.

The initial sample comprised 180 patients with COMISA, diagnosed and treated at the Center for Sleep Disorders at Haukeland University Hospital, Bergen. Unfortunately, some patients who did not fulfill the inclusion criteria were erroneously included. Thus, only 164 patients were eligible for the statistical analyses. Figure 1 shows an overview of the patient flow in the study. All patients were diagnosed following a standard respiratory polygraphic recording with type 3 portable monitors (Embletta^TM^ or NOX T3), using diagnostic criteria from the 2007 scoring manual of the American Academy of Sleep Medicine (Iber et al., 2007). Thus, apneas were defined as a reduction of ≥90% of baseline nasal airflow with duration of ≥10 s. Hypopneas were defined as a reduction of 30–90% of baseline nasal airflow with duration of ≥10 s accompanied by an oxygen desaturation of at least 4%. OSA severity was classified according to the apnea-hypopnea index (AHI): mild OSA (AHI 5-14.9), moderate OSA (AHI 15-29.9), or severe OSA (AHI ≥ 30). Insomnia was diagnosed based on the Bergen Insomnia Scale (BIS, see diagnostic criteria below). Exclusion criteria were age below 18 years and not being fluent in Norwegian.

At pretreatment, the participants completed a questionnaire including questions about age, sex, marital status, duration of sleep problems, and whether or not they had been diagnosed previously with stroke, diabetes mellitus, hypertension, angina pectoris, and depression. They also answered questions about whether or not they were currently in treatment for mental disorders and using sleep medications. In addition, weight and height were objectively measured and body mass index calculated (kg/m^2^).

Participants were consecutively randomized to either the self-help book or sleep hygiene advice in blocks of two. Information about treatment allocation was masked for the researchers until all statistical analyses were completed. Eligible patients received the written material (90 patients received the self-help book and 90 patients the sleep hygiene advice) when treatment with CPAP was initiated. CPAP was usually initiated on the day following the polygraphic recording. All patients were blinded with regard to what the other group received in terms of written material.

The primary outcome measure was insomnia severity at follow-up (scheduled at about 3 months) using the validated questionnaires the BIS (Pallesen et al., 2008) and the Insomnia Severity Index (ISI) (Bastien et al., 2001). The BIS consists of six items, and was developed based on the diagnostic criteria for insomnia according to fourth and text revision version of the Diagnostic and Statistical Manual for Mental Disorders (DSM-IV-TR) (American Psychiatric Association, 2000). The items are scored along an eight-point scale indicating the number of days per week for which a specific insomnia symptom is experienced (0–7 days). The items refer to sleep onset (sleep latency exceeding 30 min), wake after sleep onset (more than 30 min), early morning awakening (more than 30 min), non-restorative sleep, daytime impairment, and dissatisfaction with sleep. The scale is used as a continuous scale (values 0–42), where higher values indicate greater degree of insomnia, or for diagnosing insomnia. Chronic insomnia was defined as scoring 3 days per week or more on at least one of the first three items as well as 3 days per week or more on at least one of the latter two items (American Academy of Sleep Medicine, 2014). ISI consists of seven items, and is widely used as an outcome measure of insomnia severity in treatment studies (Sweetman A. et al., 2017). The scores on the ISI range from 0 to 28, where higher values indicate greater degree of insomnia. The ISI has been used in several studies on patients with COMISA (Sweetman A. et al., 2017; Philip et al., 2018).

The secondary outcome measures were CPAP adherence, sleep apnea severity (AHI), and insomnia prevalence at follow-up. CPAP adherence (defined as average nightly use in hours during the last 3 month) and AHI at follow-up were measured objectively with data from the CPAP device. Insomnia prevalence at follow-up was assessed with the BIS.

At follow-up, the participants were asked to indicate their agreement with the statement that they had read the written material (“strongly disagree,” “disagree,” “neither disagree nor agree,” “agree,” and “strongly agree”). Participants who completed follow-up were offered the written material that the other group had received – as a reward for participation. They were given no other compensation.

Written informed consent was obtained from all participants. The study was approved by the Regional Committee for Medical and Health Research Ethics (REK) in Western Norway (2014/1049/REK vest) and registered in Clinical Trials (NCT02279056) with the abovementioned primary and secondary outcome measures.

### Statistics

Power calculations were done with the G^∗^Power software version 3.1.7 (Faul et al., 2007), specifically calculating the power for within (pre vs. post)-between (book vs. sleep hygiene) interactions. A small to moderate effect size (*d* = 0.30) was expected. Alpha was set to 0.05 (two-tailed), statistical power (1 - beta) was set to 0.80 and the correlation between measures was set to 0.50. The allocation ratio between the two conditions was set to 1.0. Based on these parameters, it was estimated a total of 90 participants (45 in each condition) were needed to detect a significant group × time interaction. The Statistical Package for the Social Sciences (SPSS) version 25 was used for the data analyses. General Linear Model with repeated measures was used to compare the effect of the self-help book and the sleep hygiene advice [a 2 × 2 ANOVA with one between-group factor (self-help book vs. sleep hygiene advice) and one within-subjects/repeated measures factor (pre-treatment vs. follow-up)]. Significant interaction effects indicate different time courses for the two interventions. Paired *t*-tests, effect sizes (Cohen’s d for paired values) and Pearson chi-square tests (with continuity correction in 2 × 2 tables) were used to compare values before and after the intervention within each condition. Scores on the questionnaires in the two conditions at pre-treatment were compared with *t*-tests for independent samples and Pearson chi-square tests (with continuity correction in 2 × 2 tables), as appropriate. Intention-to-treat analyses were used, i.e., scores from pre-treatment were carried forward to the 3-month follow-up, if follow-up data were missing. Missing data were replaced for 55 patients regarding BIS, 46 regarding ISI, 37 regarding AHI, and 54 for the diagnosis of chronic insomnia (Figure 1). The same statistical analyses were also conducted on data without replacement of missing data (no intention-to-treat analyses). Furthermore, we also performed linear mixed model analyses with baseline as the time reference point. To account for clustering the model was conducted with random intercept. Mixed models allow for analyses of treatment effects even in the case of considerable attrition. Significance level was set at 0.05.

## Results

There were no significant differences between the sleep hygiene advice and self-help book groups at pre-treatment on demographic and health variables (Table 2). Although follow-up was scheduled at 3 months, the actual time for follow-up ranged from 6 to 42 weeks (mean 16.7 weeks). This was mostly due to patients re-scheduling their appointment. Among the 120 patients who responded to the question about whether they had read the written material, 6.1% (sleep hygiene advice group) and 21.8% (self-help book group) answered “disagree” or “strongly disagree.” The corresponding figures for answering “agree” or “strongly agree” were 72.3% (sleep hygiene) and 56.4% (self-help book). However, no statistical difference between the sleep hygiene advice and self-help book was found for the responses to this question (χ^2^= 7.71, *p* = 0.103).

**Table 2:** Demographics and baseline scores among patients with obstructive sleep apnea and comorbid chronic insomnia (*n* = 164).

	Sleep hygiene advice (*n* = 83)	Self-help book (*n* = 81)	*t*/χ^2^ (*df*), *p*-value
Age, mean (*SD*)	57.0 (12.1)	55.0 (11.6)	1.07 (162), 0.28^a^
Male sex	74.7%	66.7%	0.92 (1), 0.34^b^
Married/cohabiting	74.7%	66.7%	0.92 (1), 0.34^b^
Years with sleep problems (*SD*) (missing = 30)	9.9 (8.2)	10.3 (9.3)	0.29 (132), 0.77^a^
Body mass index, mean (*SD*) (missing = 1)	31.9 (5.6)	32.3 (6.0)	0.42 (161), 0.68^a^
Stroke (missing = 9)	3.9%	3.8%	<0.001 (1), 1.00^b^
Diabetes mellitus (missing = 3)	13.6%	21.3%	1.16 (1), 0.28^b^
Hypertension (missing = 2)	56.1%	45.0%	1.58 (1), 0.21^b^
Angina pectoris (missing = 11)	7.8%	5.3%	0.09 (1), 0.76^b^
Depression (missing = 6)	22.8%	20.3%	0.04 (1), 0.85^b^
In treatment for mental disorders (missing = 6)	8.8%	10.3%	<0.01 (1), 0.96^b^
Sleep medication use (missing = 4)	18.8%	21.3%	0.04 (1), 0.84^b^
BIS total score (*SD*) (missing = 11)	26.8 (7.8)	26.3 (6.3)	0.40 (151), 0.69^a^
ISI total score (*SD*) (missing = 12)	17.0 (4.0)	16.6 (4.4)	0.54 (150), 0.59^a^
AHI average score (*SD*)	24.9 (18.1)	25.6 (19.9)	0.21 (162), 0.84^a^


*^*a*^Unpaired t-tests (t). ^*b*^Pearson chi-square tests (χ^2^) with continuity correction in 2 × 2 tables. BIS, Bergen Insomnia Scale; ISI, Insomnia Severity Scale; AHI, Apnea-hypopnea-index; SD, standard deviation; df, degrees of freedom.*

Scores on the BIS and the ISI clearly improved from pre-treatment to follow-up in both the sleep hygiene advice and the self-help book groups (Table 3). However, no significant interaction effects were found. Similarly, even though the prevalence of chronic insomnia was reduced from pre-treatment to follow-up in both groups, no interaction effect was found (χ^2^= 0.66, *p* = 0.42). In the group that received the sleep hygiene advice, 21.7% no longer had chronic insomnia at follow-up, whereas the corresponding figure for the self-help book group was 28.4%. Furthermore, there was no significant interaction when analyzing AHI (Table 3) or CPAP adherence [mean adherence according to the CPAP device: 2:54 h (sleep hygiene) and 2:55 h (self-help book), independent *t*-test *p* = 0.98].

**Table 3:** Effects of a self-help book for insomnia compared to sleep hygiene advice among patients with obstructive sleep apnea and comorbid chronic insomnia concurrently being treated with continuous positive airway pressure (CPAP).

	Sleep hygiene advice (*n* = 83)			Self-help book (*n* = 81)			
	Mean (*SD*) Pre – Post	*t* (*df*), *p*-value^a^	Effect size^b^	Mean (*SD*) Pre – Post	*t* (*df*), *p*-value^a^	Effect size^b^	*F* (*df*), *p*-values^c^
**Intention-to-treat**							
BIS	26.8 (7.8) – 21.8 (10.1)	4.63 (80), <0.001	0.55	26.3 (6.4) – 22.4 (8.5)	4.78 (71), <0.001	0.52	0.57 (1,151), 0.45
ISI	17.0 (4.0) – 14.1 (5.8)	5.02 (73), <0.001	0.58	16.6 (4.4) – 13.6 (5.5)	6.33 (77), <0.001	0.60	0.04 (1,150), 0.84
AHI^d^	24.9 (18.1) – 7.9 (14.6)	9.41 (82), <0.001	1.03	25.6 (19.9) – 8.7 (16.4)	8.23 (80), <0.001	0.93	0.01 (1,162), 0.94
**No intention-to-treat^e^**							
BIS	27.1 (7.6) – 19.9 (10.3)	4.93 (55), <0.001	0.80	25.2 (6.1) – 18.5 (7.7)	5.27 (41), <0.001	0.96	0.05 (1,96), 0.82
ISI	17.2 (3.7) – 13.1 (5.9)	5.38 (52), <0.001	0.83	16.2 (4.4) – 11.7 (5.0)	7.27 (52), <0.001	0.96	0.21 (1,104), 0.65
AHI^d^	24.2 (16.9) – 3.1 (5.0)	10.91 (66), <0.001	1.69	24.6 (18.8) – 1.9 (1.8)	9.79 (59), <0.001	1.70	0.30 (1,125), 0.59


*^a^Paired t-test, comparing values before and after the intervention (separate tests for the self-help book and the sleep hygiene advice). ^b^Effect size (Cohen’s d) for paired values. ^c^General Linear Model with repeated measures (a mixed between-within subjects 2 × 2 ANOVA) comparing the effect of the self-help book and the sleep hygiene advice. ^d^AHI post-treatment was measured with data from the CPAP device. ^e^In these analyses the total number of patients varied between 98 (BIS), 106 ISI and 127 (AHI). BIS, Bergen Insomnia Scale; ISI, Insomnia Severity Index; AHI, apnea-hypopnea-index.*

Non-significant findings between the two groups were also found when we analyzed data without intention-to-treat (Table 3) and when we analyzed with linear mixed models [BIS, group × time: *F* = 0.35(*df* 1,138.7), *p* = 0.56; ISI, group × time: *F* = 0.90(1,127.8), *p* = 0.35; AHI, group × time: *F* = 0.32(1,112.5), *p* = 0.57]. Furthermore, when excluding patients without sleep onset problems (39%) in the intention-to-treat analyses, we still did not find any interaction effects on the BIS [*F* = 1.15(*df* 1,90), *p* = 0.29], ISI [*F* = 0.11(1,92), *p* = 0.74], or AHI [*F* = 0.17(1,97), *p* = 0.68].

## Discussion

The self-help book was not more effective than sleep hygiene advice in improving insomnia symptoms, increasing CPAP adherence, or reducing AHI during CPAP treatment. Thus, our initial hypothesis was not supported. However, both patient groups clearly improved their sleep, and as many as 22–28% of the patients no longer fulfilled the criteria for chronic insomnia at follow-up.

There are many possible reasons why the self-help book did not improve the insomnia symptoms more than the sleep hygiene advice. These patients were referred to the hospital with suspicion of OSA, indicating that insomnia was not their main complaint. Thus, even though the self-help book is shown to significantly improve sleep compared to sleep hygiene advice among patients with chronic insomnia (Bjorvatn et al., 2011), patients with COMISA will likely be less motivated for such self-help treatment. This is also in line with the present findings showing that only 56.4% of the patients in the self-help book group agreed to have read the book. Another point to make is that all patients in the present study initiated CPAP treatment at the same time as the written material was handed out. If CPAP improved the patients’ sleep and well-being from day one, the patients may not feel they need any other treatment for their sleep complaints. Studies have also shown that most such patients prefer to initiate CPAP first as adhering to two new treatments at once may be too overwhelming (Ong et al., 2017).

Both patients in the sleep hygiene advice and self-help book groups reported significantly better sleep at follow-up. The most likely explanation is that CPAP improved sleep through reducing apneas and hypopneas. It is well-known that OSA may disturb sleep and increase the number of nightly awakenings (Krakow et al., 2001; Smith et al., 2004). If OSA is the cause of the insomnia symptoms, CPAP will likely be an effective solution. In line with our findings, other studies have shown that CPAP reduces insomnia symptoms (Bjornsdottir et al., 2013; Glidewell et al., 2014). However, even though sleep maintenance problems may be caused by OSA, sleep onset problems are less likely to improve with CPAP (Bjornsdottir et al., 2013). Still, our results remained the same also when only patients with sleep onset problems were included in the analyses. Furthermore, our data indicated that CPAP adherence was not very high, questioning whether the improvement in sleep can be explained by CPAP alone. Another explanation for improved sleep in both patient groups may be regression-to-the-mean, which means that patients tend to improve over time no matter what treatment they receive (Yu and Chen, 2014). Patients usually commence in studies when their complaints are peaking, and at follow-up, symptoms are often reduced spontaneously. A third explanation for improved sleep at follow-up may be that the control group received efficient sleep hygiene advice. Doctors/therapists often give similar advice for the treatment of sleep problems (Stepanski and Wyatt, 2003; Morgenthaler et al., 2006). Thus, the sleep hygiene advice group may have inflated the possibility of finding a significant effect of the self-help book. However, the effect of such simple advice is questionable, and current guidelines do not recommend sleep hygiene as monotherapy for insomnia (Morgenthaler et al., 2006). Furthermore, our previous randomized controlled trial among patients with chronic insomnia had similar treatment options, and in that study we found clear differences between the groups in favor of the self-help book compared to the sleep hygiene advice (Bjorvatn et al., 2011). Still, we do not know if similar improvement in insomnia symptoms would have been found if the control group had received no treatment or treatment as usual, or if the treatment had taken place in sequence rather than in parallel, as in the present study (Ong et al., 2017).

Many people are affected by OSA, and many have comorbid insomnia (Luyster et al., 2010; Bjornsdottir et al., 2012, 2013; Bjorvatn et al., 2015; Sweetman A.M. et al., 2017). Two recent studies showed that insomnia symptoms were associated with early quitting of CPAP treatment (Eysteinsdottir et al., 2017) and poorer CPAP use at 6 month follow-up (Wallace et al., 2018), clearly indicating that addressing insomnia symptoms is important in patients with OSA. However, assessment and treatment of insomnia are not common in clinics focusing on sleep-related breathing disorders, like OSA. Thus, there is a need for an easily available insomnia treatment option for COMISA-patients. A self-help book would be an appropriate low-threshold treatment option, but our study suggests that sleep hygiene advice may be just as effective for this patient group. However, as detailed below, our study suffers from major limitations impeding us to make firm conclusions. Further studies on the role of CBTi among OSA patients with comorbid chronic insomnia are therefore warranted.

Our study has major limitations and some strengths that should be noted. A strength was that this was a randomized controlled double-blind trial comparing two active treatments. Another strength was that the insomnia questionnaires used were well validated. The use of intention-to-treat analyses improves the generalizability of the results, and we also ran mixed model analyses and other statistical analyses, in order to strengthen our interpretation of the data. Furthermore, use of objective CPAP adherence and AHI data eliminated the risk of common method bias, when analyzing these data against self-reports provided by the patients. A major limitation was the large attrition rate and large number of missing values. Some of the included patients did not fulfill the inclusion and exclusion criteria, and had to be excluded before statistical analyses (e.g., 10 did not have chronic insomnia, 1 did not have OSA, 1 was already using CPAP). Many patients did not answer all items in the self-report questionnaires. This was handled with replacing missing values with scores from pre-treatment (intention-to-treat), but such a procedure is conservative and warrants caution when interpreting the data. Furthermore, even though follow-up was scheduled at 3 months, the time interval between pre- and follow-up assessment varied considerably. We decided to exclude patients attending follow-up more than 1 year following treatment completion (three patients), but still, for some of the included patients the follow-up assessment was a lot later than anticipated. This was mainly due to patients re-scheduling their appointment. How this may have influenced the results is unclear. Another limitation was related to the AHI assessments. We used polygraphy for assessing AHI at pre-treatment, whereas AHI was assessed from the CPAP device at post-treatment. Thus, at post-treatment, AHI was assessed only when CPAP was in use, thereby explaining the low values at follow-up. If polygraphy had been used at follow-up, AHI would likely be higher, since some patients are not compliant with CPAP throughout the night. Whether or not the patients adhered to the advice given in the written material was only retrospectively assessed, and this represents a major limitation of the study as poor adherence may explain why the self-help book was not superior to the sleep hygiene advice. Furthermore, we did not include a true control group that did not receive any advice concerning their sleep problem, and this may be considered as a limitation. However, in terms of clinical trials, to include an active control group (as in the present study) is regarded as more rigorous scientifically since such a control condition adjusts for the effect of believing to be treated for benefit.

## Conclusion

To conclude, the self-help book did not improve insomnia symptoms, increase CPAP adherence, or reduce AHI more than what was detected in the sleep hygiene advice condition. The book can therefore not be recommended as a first-line treatment for patients with COMISA. Both patient groups showed clearly improved sleep, and this may be caused by the CPAP device itself, as an effect of the sleep hygiene advice *per se*, or other factors such as regression toward the mean.

## Author Contributions

BB designed the study, analyzed the data, and wrote the paper. TB collected and interpreted the data, and revised the paper. SL and SP designed the study, interpreted the data, and revised the paper. IS designed the study, analyzed the data, and revised the paper.

## Conflict of Interest Statement

BB is the author of the self-help book. The remaining authors declare that the research was conducted in the absence of any commercial or financial relationships that could be construed as a potential conflict of interest.
